# Combatting anthrax outbreaks across Nigeria’s national land borders: need to optimize surveillance with epidemiological surveys

**DOI:** 10.1186/s40249-024-01179-3

**Published:** 2024-02-01

**Authors:** Hammed O. Mogaji, Babatunde Adewale, Stella I. Smith, Ehimario U. Igumbor, Chidumebi J. Idemili, Andrew W. Taylor-Robinson

**Affiliations:** 1 Yale School of Public Health, Yale University, New Haven, USA; 2 Parasitology and Epidemiology Unit, Federal University Oye-Ekiti, Oye-Ekiti, Nigeria; 3 Department of Public Health and Epidemiology, Nigerian Institute of Medical Research, Lagos, Nigeria; 4 Department of Molecular Biology and Biotechnology, Nigerian Institute of Medical Research, Lagos, Nigeria; 5 Centre for Infectious Disease Research, Nigerian Institute of Medical Research, Lagos, Nigeria; 6 Department of Public Health, Walter Sisulu University, Mthatha, South Africa; 7 Institute of Health Policy, Management, and Evaluation, University of Toronto, Toronto, Canada; 8 College of Health Sciences, Vin University, Gia Lam District, Hanoi, Vietnam; 9 Center for Global Health, Perelman School of Medicine, University of Pennsylvania, Philadelphia, USA

**Keywords:** Anthrax, *Bacillus anthracis*, Zoonosis, Livestock, Pastoralist, Outbreak, Surveillance, Control, Prevention, Nigeria

## Abstract

**Background:**

Anthrax is a non-contagious zoonotic disease caused by the Gram-positive, spore-forming bacterium *Bacillus anthracis*. Infection is common in livestock and wild animals such as cattle, goats, sheep, camels, and antelopes. In humans, anthrax may occur after contact with contaminated carcasses or animal products like milk and meat. The best method to prevent anthrax in people is to ensure livestock are vaccinated, which significantly limits the risk of zoonotic spread to humans. However, the rate of vaccination of domesticated animals kept by nomadic pastoralists in West Africa is low. These groups regularly cross over national boundaries with their grazing herds. Nigeria is a country that historically has done comparatively well to contain this public health threat. However, in 2023 several outbreaks of human disease appear linked to the consumption of anthrax-contaminated animal products brought into Nigeria by pastoralists from neighboring countries. Clinical manifestations include skin sores or ulcers, nausea, vomiting, and fever. This article aims to raise awareness of recent outbreaks of anthrax in West Africa and to call for a renewed focus on measures to combat this neglected public health concern to the region.

**Main body:**

The imperative to pinpoint pivotal issues relating to the ongoing emergence of anthrax cases in Nigeria cannot be overstated. By delving into the prevalence of anthrax in both livestock and human populations residing along Nigeria’s borders, unraveling the genetic diversity and potential sources of *B. anthracis* strains, and identifying the primary animal host(s) responsible for transmission, we stand to enhance our understanding of this critical issue. Furthermore, investigating the multifaceted factors contributing to anthrax transmission, assessing community knowledge and practices, mapping common migratory routes of pastoralists, and formulating targeted intervention strategies tailored to the challenges of border communities, are each crucial steps towards effective control and prevention.

**Conclusion:**

Closing these knowledge gaps on anthrax is not only essential for safeguarding both animal and human health but also for fostering sustainable and resilient communities. Addressing research questions on these interdisciplinary concerns will undoubtedly pave the way for informed decision-making, proactive measures, and a more secure future for Nigeria and its border regions.

**Graphical Abstract:**

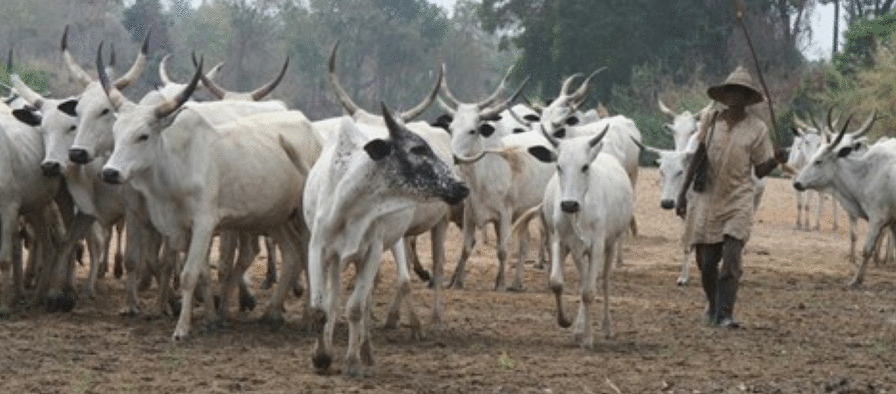

## Background

Zoonotic diseases, which affect both humans and animals, can impose a significant burden on human and veterinary healthcare systems, especially in countries with limited resources [1]. Among these zoonoses, anthrax looms as a prominent concern, demanding increased public health attention. It is a rare but, if not treated, frequently deadly disease. An anthrax spore vaccine is available that is recommended for livestock at high risk of infection or those grazing in areas where previous infections have occurred. Anthrax is caused by the Gram-positive, rod-shaped bacterium *Bacillus anthracis*, a spore-forming obligate pathogen that primarily affects herbivores. From an anthropocentric perspective, this notably includes livestock. The bacterium exists in two forms: dormant spores found in the environment; and the vegetative state that infects hosts [2]. The spores are highly resilient and can persist in the environment, particularly in soil or on plant leaf litter contaminated with anthrax spore-loaded necrophagous fly feces at the site where a deceased infected host’s carcass is found [3]. However, vegetative forms of the bacterium may also gain entry to the human body through direct contact, such as when a person consumes meat from an infected animal or handles carcasses, hides, or bones. In endemic areas, necrophagous blowflies have also been identified as potential vectors for transmitting the pathogen [4]. This can be either by non-biting flies depositing bacilli or spores in open cuts or abrasions, or by biting flies carrying *B. anthracis* in infected blood on their mouthparts when feeding.

### Clinical forms of anthrax

The manifestation of anthrax illness a person develops depends on how the bacterium enters the body primarily through one of three routes, via the skin, lungs, or gastrointestinal system [5]. If left untreated with antibiotics, all forms of anthrax can spread throughout the body and potentially lead to death. The most common manifestation is cutaneous anthrax, which is usually the least dangerous. It occurs when anthrax spores enter the skin, typically through minor cuts or scrapes, often from handling infected animals or their products [5, 6]. Cutaneous anthrax primarily affects the head, neck, forearms, and hands. Without medical intervention, up to 20% of cases can be fatal, but with appropriate antibiotic treatment nearly all patients survive [5]. Inhalation anthrax is the deadliest form, typically resulting from breathing in *B. anthracis* spores [7]. It can affect individuals working in settings like wool mills, slaughterhouses, or tanneries. Inhalation anthrax starts in the chest’s lymph nodes and can lead to severe breathing problems and shock. Without treatment, it is almost always fatal, but aggressive treatment increases survival to around 55% [5]. Hence, *B. anthracis* is classified as a tier 1 biological agent and toxin. This classification indicates that anthrax poses a significant risk of intentional misuse, with the potential for causing mass casualties, substantial economic impact, harm to critical infrastructure, and erosion of public confidence [8]. Gastrointestinal anthrax occurs following consumption of undercooked and raw meat from an infected animal. It can affect the upper gastrointestinal tract, stomach, and intestines. More than half of cases are fatal, but with proper treatment, 60% of patients survive [5, 9]. In addition, injection anthrax is a unique type that has been reported among communities of intravenous drug users, mostly in western and northern Europe (UK, France, Germany, Denmark, Norway), who inject heroin diluted with bone meal contaminated with anthrax spores [10]. Symptoms may resemble cutaneous anthrax but can spread more quickly and be harder to identify and treat [5, 10]. Notably, other common bacteria can cause similar skin or injection site infections, so anthrax is not the sole cause to consider in these cases [5].

### Historical context and epidemiology

Historically, anthrax has been one of the foremost causes of uncontrolled mortality in cattle, sheep, goats, horses and pigs worldwide, with humans invariably contracting infection directly or indirectly from these animals [2]. Currently, it is estimated that the disease causes up to 100,000 cases in cattle and other livestock annually worldwide, with approximately 1.8 billion people placed at direct risk by virtue of residing in anthrax-prone areas [11]. An accurate global measure of the anthrax burden is still emerging [12]. Nevertheless, the disease is persistently underreported, and adjudged to be common in some Mediterranean countries, in small pockets in Canada and the USA, certain countries of Central and South America and in Central Asia, several sub-Saharan African nations, and in western China. However, there are also sporadic cases and outbreaks reported elsewhere [4]. A more recent review posited that anthrax incidence varied widely between countries, ranging from 0.03 to 1.4 per 100,000 inhabitants in Ghana and Georgia, respectively [12]. Anthrax transmission is influenced by a multitude of environmental factors, including fluctuations in pH level of spore-contaminated soil, variations in temperature, water availability, and cation concentrations of contaminated soil. Additionally, seasonality and the availability of pasture for livestock grazing play a significant role, along with the health status of the animal host, the abundance of insect populations, human activities including but not limited to reliance on imported food products [2, 4, 13].

**Fig. 1:**
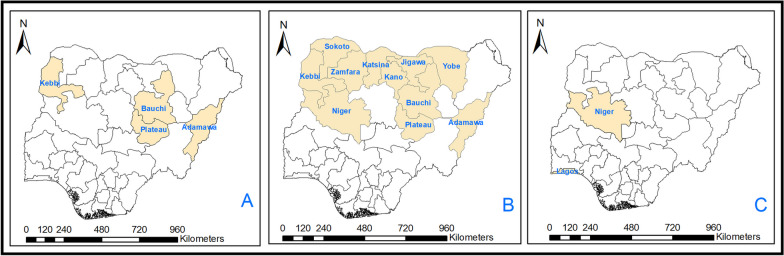
Map of Nigeria showing the administrative areas where *Bacillus anthracis* has been reported or predicted in Nigeria. **A** Administrative areas with historical data and available genomic data on *B. anthracis* in Nigeria. **B** Administrative areas modelled to be at high risk of *B. anthracis* in Nigeria [1]. **C** Administrative areas where the 2023 outbreak of *B. anthracis* was reported in Nigeria

Anthrax outbreaks persistently afflict Africa, impacting both animal and human populations. During the past decade, several countries including Benin, Burkina Faso, Ghana, Niger, and Togo in West and Central Africa have experienced recurring episodes of anthrax [4, 14]. Each of these nations is a near neighbor of Nigeria, and their outbreaks have occurred predominantly between January and May, coinciding with the transition from the dry season to the onset of the wet season. Anthrax outbreaks in Nigeria have a history linked to cattle and were believed to have been eliminated in 2004 until their reappearance in 2023 [15]. While there is limited historical data on the distribution of anthrax in Nigeria, existing reports suggests that it could be found in the north-central part of the country where significant outbreaks have occurred previously [15]. This region encompasses the northern core states of Nigeria, which have traditionally been a major source of the country’s meat supply [16], hence are classified as high-risk areas (Fig. 1). Available information confirms that the genetic diversity of *B. anthracis* in Nigeria belongs to a distinct West African genetic clade, i.e. Aβ or E, like those found in neighboring Cameroon, Chad, and Mali [1]. The genetic similarities of the bacterium across these regions may be a result of historical trade patterns and ongoing nomadic pastoralism [17], which facilitate the spread of a well-adapted strain complex.

The persistence of anthrax within this subregion has been attributed in part to factors specific to the pathogen’s survival in the soil, prevailing environmental conditions, inadequate vaccination services and absence of proper disposal methods for infected animal carcasses [4]. Socio-cultural practices at the community level, such as the slaughter of sick animals or the butchering of dead animals for salvage purposes, as well as the consumption or handling of meat from infected animals, contribute to recurrent cases of anthrax in humans. Recent reports have indicated the reduced likelihood of anthrax outbreaks in neighboring countries such as Cameroon and Chad, which share borders with Nigeria [1, 18, 19]. Therefore, the recent outbreaks in the northern part of Ghana and more recently within Nigeria itself underscore the immediate requirement for comprehensive actions that are not limited to surveillance, livestock vaccination, and appropriate disposal of animal carcasses. This applies particularly to the Nigerian border states where nomadic farming practices are prevalent [20, 21].

### Resurgence of anthrax in Nigeria

Based on available surveillance data sourced from the World Animal Health Information System run by the World Organisation for Animal Health, an ongoing outbreak of anthrax has been identified, spanning three distinct locations (Suleja, Marina and Oko-Oba) across two administrative units (Niger and Lagos States) within the country (Fig. 1; Table 1). These administrative areas were previously notified (Niger) or predicted to be at a high risk of anthrax (Lagos) (Fig. 1). Confirmed positive samples were identified using bacterial culture, and the positivity rate was reported as the percentage of total diagnoses that tested positive for anthrax among susceptible livestock. The initial outbreak was reported in Niger on 27 June 2023, affecting 173 susceptible older animals on a farm with a test positivity rate of 20%. A subsequent outbreak emerged in the southwestern region of the country, specifically in a backyard in Lagos Marina, with an incidence rate of 52.6% among freshly exposed animals. The most recently reported outbreak took place in Oko-Oba, within a slaughterhouse setting, where a test positivity rate of 0.3% was recorded among 999 new livestock (Table 1). This outbreak prompted veterinary and public health authorities to examine a sample of 1201 susceptible live animals across the three locations, with the majority (85.6%) being recently introduced from locations outside Nigeria. The incidence of anthrax in this population was estimated to be 4.0%, exhibiting a notable variation between older animals (47.9%) and newly introduced younger ones (1.3%) (Table 2). The overall mortality rate associated with the outbreak was estimated at 66.7%, with similar trends observed among both older animals (65.7%) and those newly introduced (69.2%). A mere 18.8% of the animals that tested positive were culled and their carcasses disposed of, while others were sacrificed and sold for domestic purposes, which clearly raises public health concerns [22]. Also, none of the examined animals had been vaccinated against anthrax. Analyzing the data by livestock species and type, the highest incidence and mortality rates were documented among newly introduced sheep, followed by cattle. In contrast, camels demonstrated no anthrax-associated mortality, with only one case recorded among both old and new livestock. Additionally, no cases or deaths were observed in either the old or new goat groups, suggesting their lack of susceptibility. Overall, these findings emphasize the urgency of implementing targeted interventions to curb disease transmission, especially within cattle and sheep populations displaying higher susceptibility and mortality rates.

**Table 1:** Overview of anthrax outbreaks in 2023 across administrative areas of Nigeria

Date of outbreak	Administrative area	Location	Coordinates	Epidemiological Unit	Species	New livestock	Number (%)		
							Susceptible	Cases^a^	Deaths^b^
27 June 2023	Niger	Gajiri, Suleja	9.244089 N, 7.207087 E	Farm	Sheep	No	73	15 (20.5)	10 (66.7)
						Yes	0	0 (0)	0 (0)
					Cattle	No	100	20 (20.0)	13 (65.0)
						Yes	0	0 (0)	0 (0)
16 July 2023	Lagos	Marina	6.44475 N, 3.39951 E	Backyard	Camel	No	0	0 (0)	0 (0)
						Yes	1	1 (100)	0 (0)
					Goat	No	0	0 (0)	0 (0)
						Yes	4	0 (0)	0 (0)
					Cattle	No	0	0 (0)	0 (0)
						Yes	10	0 (0)	0 (0)
					Sheep	No	0	0 (0)	0 (0)
						Yes	14	9 (64.3)	9 (100)
18 July 2023	Lagos	Oko-Oba	6.64978 N, 3.32068 E	Slaughterhouse	Cattle	No	0	0 (0)	0 (0)
						Yes	999	3 (0.3)	0 (0)

^a^Number (proportion) of domestic animals that tested positive for *Bacillus anthracis*; ^b^Number (proportion) of domestic animals that tested positive for *B. anthracis* and are dead. Data provided by the World Organisation for Animal Health [taken from reference [22] and online updates]

**Table 2:** Overview of anthrax outbreaks in Nigeria, June–July 2023, by species and type of animal

		Number of domestic animals (%)						
Species	Livestock	Susceptible	Cases^a^	Deaths^b^	Killed and disposed^c^	Slaughtered for commercial use^c^	Vaccinated^c^	
Camel	Old	0	0	0 (0)	0 (0)	0 (0)	0 (0)	
	New	1 (100)	1 (100)	0 (0)	0 (0)	0 (0)	0 (0)	
	*Total*	*1 (100)*	*1 (100)*	*0 (0)*	*0 (0)*	*0 (0)*	*0 (0)*	
Cattle	Old	100 (9.0)	20 (20.0)	13 (65.0)	0 (0)	7 (100)	0 (0)	
	New	1009 (91.0)	3 (0.3)	0 (0)	3 (100)	0 (0)	0 (0)	
	*Total*	*1109 (100)*	*23 (2.1)*	*13 (56.5)*	*3 (30.0)*	*7 (70.0)*	*0 (0)*	
Goat	Old	0 (100)	0 (0)	0 (0)	0 (0)	0 (0)	0 (0)	
	New	4 (100)	0 (0)	0 (0)	0 (0)	0 (0)	0 (0)	
	*Total*	*4 (100)*	*0 (0)*	*0 (0)*	*0 (0)*	*0 (0)*	*0 (0)*	
Sheep	Old	73 (83.9)	15 (20.5)	10 (66.7)	0 (0)	0 (0)	0 (0)	
	New	14 (16.1)	9 (64.2)	9 (100)	0 (0)	0 (0)	0 (0)	
	*Total*	*87 (100)*	*24 (27.6)*	*19 (79.2)*	*0 (0)*	*0 (0)*	*0 (0)*	
Overall	Old	173 (14.4)	35 (47.9)	23 (65.7)	0 (0)	7 (58.3)	0 (0)	
	New	1028 (85.6)	13 (1.3)	9 (69.2)	3 (75.0)	0 (0)	0 (0)	
	* Total *	* 1201* *(100) *	* 48* *(4.0) *	* 32* *(66.7) *	* 3* *(18.8) *	* 7* *(21.9) *	* 0* *(0) *	

^a^Number (proportion) of domestic animals susceptible to *Bacillus anthracis*; ^b^Number (proportion) of domestic animals tested positive for *B. anthracis*; ^c^proportion of domestic animals tested positive for *B. anthracis* and still alive. Data provided by the World Organisation for Animal Health [reference [22] and online updates]

Evidently, anthrax has emerged as a disease of significant veterinary and public health concern owing to outbreaks in neighboring countries (Benin, Burkina Faso, Cameroon, Chad, Ghana, Niger, and Togo) that have boundaries with Nigeria, and more recently within Nigeria in Niger and Lagos States [21, 23, 24]. Most of the international trade between Nigeria and these fellow African nations revolves around livestock farming by the Fulani population, which is recognized as the largest nomadic ethnic group globally [25]. These nomadic communities rely heavily on livestock for sustenance and income, pursuing a lifestyle that follows a seasonal migratory cycle which crosses national borders in search of sufficient pasture and water resources for their herds [26, 27]. The practice of transhumance herding, prevalent in many regions of Nigeria and neighboring countries, has raised concerns regarding the potential dissemination and lasting presence of zoonotic diseases. Available reports suggest that this movement pattern is linked to the continued existence of *Rhipicephalus* (*Boophilus*) *microplus*, known for transmitting *Babesia* parasites among cattle [28], as well as the distribution of snail intermediate hosts responsible for *Schistosoma* infections in livestock [29]. This traditional herding pathway may also serve as a potential conduit for the spread of *B. anthracis*. In addition to the close proximity to livestock, the reliance on livestock products, including consumption of improperly roasted bush-meat known as “suya” or “kilishi”, is a common practice among the Nigerian population that heightens the risk of contracting anthrax.

### Molecular diagnostics required

Our understanding of the genetic diversity of *B. anthracis* is still evolving [30, 31], and the available evidence from Nigeria is both limited and outdated [1]. Recent historical genomic data indicates the presence of a distinct West African genetic clade circulating in Nigeria [1]. However, during the 2023 outbreak, anthrax diagnostics relied on bacterial culture, presenting a significant limitation, as the outbreak might have been caused by a different species, possibly *Bacillus cereus* biovar anthracis, endemic in West Africa and which causes anthrax-like disease [32]. The adoption of more refined molecular approaches becomes crucial to accurately identify the etiological agent and to decipher the genetic diversity of such species. The absence of genomic information poses a challenge for a more robust phylogenomic analysis. The speculation that West Africa might be a hotspot for anthrax evolution with potential connections to Europe and possibly the Americas can only be evaluated using genomic data. Furthermore, understanding circulating genotypes is essential for detecting longitudinal changes, such as when a previously predominant isolate is replaced by an imported one. This knowledge is key for identifying which neighboring countries or states within Nigeria contribute the most to these influxes. Therefore, a comprehensive genomic analysis would play a pivotal role in implementing more specific countermeasures.

### Interdisciplinary action required

Effectively addressing anthrax outbreaks in Nigeria therefore requires comprehensive strategies that emphasize understanding the current transmission status, animal movement patterns, and the broader socio-economic context in which pastoral herders operate. Epidemiological surveys employing more refined molecular diagnostic approaches are required for identifying circulating strains and investigating phylogenetic linkages. Additionally, these surveys could be complemented by conducting serological assays that examine previous exposure and associated risk factors among animals and human populations living in communities situated along the borders of Nigeria, or locations where past outbreaks have occurred. Such research is important to enhance understanding of local contexts driving pathogen transmission and/or disease outbreak, prepare for future outbreaks and to identify the contributing factors facilitating transmission. These studies may also incorporate mathematical or statistical modeling techniques, including the development of spatial models using geographic information systems to map endemic areas and the incorporation of various parameters related to disease spread through agent-based modeling. Additionally, a compartmental model based on systems of ordinary differential equations, consisting of different classes of susceptible, infectious, or recovered models for spread of disease, would be valuable for assessing anthrax transmission dynamics, intervention strategies, and cost-effectiveness, particularly in developing countries like Nigeria. This information will also provide crucial insights to guide formulation of focused initiatives designed to control and prevent anthrax outbreaks in Nigeria, thus limiting livestock losses and reducing human disease.

## Conclusions

In order to control the escalation of anthrax cases in Nigeria, a multifaceted approach based on available surveillance data is imperative. Efforts should be directed towards curbing the disease in cattle, with a particular focus on national border districts. Community awareness interventions under a One Health approach should be instigated to educate pastoralist communities and livestock market traders on anthrax control and prevention. Enhancing transmission surveillance, strengthening zoonotic outbreak responsiveness, and widening access to diagnostic tools, especially during outbreak seasons, each contribute to limiting anthrax cases in both animals and humans, thereby reducing associated fatalities, illnesses, and economic losses. Moreover, rigorously enforced regulation of cross-border livestock traffic is challenging but desirable to reduce, if not prevent, anthrax transmission. Recommendations include preemptive anthrax vaccination for domestic ruminants, increased monitoring for sudden livestock deaths, proper carcass disposal, and raised capacity for microbiology laboratory case confirmation. Drawing on the paramount role of livestock vaccination in reducing human cases, as well as understanding the overlap of pathogen and local host habitat selection, is key to effective anthrax management in Nigeria and the entire West Africa region.

## Data Availability

Not applicable.
